# Evaluation of SLOG/TCI-III pediatric system on target control infusion of propofol

**DOI:** 10.1186/1479-5876-9-187

**Published:** 2011-11-01

**Authors:** Wan-hua Yang, Hong-bin Gu, Bing Chen, Juan Li, Qiu-wei Fan, Yong-fang Yuan, Xiangdong Wang

**Affiliations:** 1 Department of Pharmacy, Ruijin Hospital, Shanghai Jiao Tong University School of Medicine, Shanghai 200025, China; 2 Department of Anesthesiology, Shanghai Children′s Medical Centre, Shanghai Jiao Tong University School of Medicine, Shanghai 200127, China; 3 Department of Anesthesiology, Ruijin Hospital, Shanghai Jiao Tong University School of Medicine, Shanghai 200025, China; 4 Department of Pharmacy, The Third Hospital, Shanghai Jiao Tong University School of Medicine, Shanghai 201900, China; 5 Department of Respiratory Medicine, Fudan University Zhongshan Hospital, China

**Keywords:** Propofol, plasma concentration, high performance liquid chromatography, drug delivery system, pediatric, evaluation

## Abstract

**Background:**

The target-controlled infusion-III (SLOG/TCI-III) system was derived from a model set up by the local pediatric population for target control infusion of propofol.

**Methods:**

The current study aimed at evaluating the difference between target concentrations of propofol and performance, which was measured using the SLOG/TCI-III system in children. Thirty children fulfilling the I-II criteria according to American Society of Anesthesiology were enrolled in the study. The target plasma concentration of propofol was fed into the SLOG/TCI-III system and compared with the measured concentrations of propofol. Blood samples were collected and analyzed by high performance liquid chromatography with fluorescence detector. The performance error (PE) was determined for each measured blood propofol concentration. The performances of the TCI-III system were determined by the median performance error (MDPE), the median absolute performance error (MDAPE), and Wobble (the median absolute deviation of each PE from the MDPE), respectively.

**Results:**

Concentration against target concentration showed good linear correlation: concentration = 1.3428 target concentration - 0.2633 (r = 0.8667). The MDPE and MDAPE of the pediatric system were 10 and 22%, respectively, and the median value for Wobble was 24%. MDPE and MDAPE were less than 15 and 30%, respectively.

**Conclusions:**

The performance of TCI-III system seems to be in the accepted limits for clinical practice in children.

## Introduction

The effectiveness and safety of pharmacokinetics and pharmacodynamics of anesthetic drugs, such as propofol, were studies in the pediatric population. The application of target controlled infusion (TCI) has been expanded to pediatric anesthetization and sedation during surgery, as a functional pharmacokinetic model primarily for propofol and Alfentanil. Clinical studies on TCI of propofol for pediatric anesthetization have verified that factors such as ethnic group, body weight, and age influence pharmacokinetic parameters, which can vary greatly from those of adults [1-3]. The distributional volume of central compartments in pediatrics is larger than the corresponding value in adults. As the Diprifusor software was written based on adult parameters, Absalom *et al*. pointed out that the adult parameters of Marsh model might not be appropriate for TCI of propofol in children [4]. This challenge was also emphasized in TCI of propofol to children in pediatric anesthetization [5].

"Paedfusor" was introduced as the first TCI system for pediatric induction and maintenance of anesthesia, followed by the analytic research on pharmacokinetic parameters of propofol in adults and children, associated with age, body weight, samples, and drug administration approach [6]. The difference in the pharmacokinetic model of propofol for adults lies in the fact that the central compartmental volume is expanded correspondingly to the ratio of infantile ages by Paedfusor. The performance of TCI for cardiac catheterization or selective cardiac surgery was studied in 29 children between 1 and 15 years old using the Paedfusor system [6]. The median performance error, median absolute performance error of bias and precision of the predicative system were about 4 and 10%, respectively [6]. The degree of Wobble was about 8%. In comparison to the acquired pediatric values with "Diprifusor" for adults, these results are more accurate and satisfactory in clinical effect. In China, the current practice is the use of embedded TCI systems that are either calibrated according to the adult pharmacokinetic parameters or according to western pediatric data.

The accuracy of such practice is still questionable, due to a number of influencing factors such as ethnicity, age, drug disposition and drug response. The SLOG/TCI-III system is based on pharmacokinetic characteristics derived for the local pediatric population. The president study aimed at evaluating the quality analysis of equivalence in the actual measured concentration and the target concentration of propofol using the TCI-III in pediatric patients with surgery. We hope our preliminary results can attract the special attention to standardize the performance and clinical application of the pediatric model for target-controlled infusion of propofol. The present study also indicates needs of investigations to explore cellular and molecular mechanisms and system-specific biomarkers as a bed to bench approach by integrating clinical bioinformatics [7-9].

## Materials and methods

### Primary equipments

Waters high performance liquid-phase chromatography with the inclusive of constant flow pump of model 510, fluorescence testing instrument of 2475, and spectrum workstation of Millennium2010, and chromatographic column Zorbax Eclipse C18 with 250 mm × 4.6 mm, 5 μm, were used. HP 78352 multi-function monitor (Qindao subsection of HP company, China) and monitoring instrument for bi-spectral index (Covidien, Ireland) were used. Injection pump of model TCI-III with included parameters of pediatric propofol was purchased from the Beijing SLOG/TCI-III Technology Development Co., (China).

### Primary test agents

The testing agents were used, e.g. propofol (99.9% purity, Astrazeneca, UK), thymol (batch no. 100508-2003.1, National Institute for the Control of Pharmaceutical and Biological Products, China), acetonitrile (high performance liquid chromatography grade, AS-1122, TEDIA, USA), methanol (high performance liquid chromatography grade, MS-1922, TEDIA), glacial acetic acid (T2004D32G, Sinopharm Chemical Reagent Co., Shanghai, China), 20% trichloroacetic acid & 20% perchlorate, blank plasma (plasma of healthy subjects, provided by Shanghai Ruijin Hospital, China), sodium lactate & Ringer's solution (2007060102, HuaYu Pharmaceutical Co., Wuxi, China), injection of propofol (Diprivan) (Imported drug registration H20030427, FA283, Imported drug registration H20030481, FB908, Astrazeneca, Italy), injection of midazolam (20061204, Jiangsu Nhwa Pharma. Co., Jiangsu, China), ketamine hydrochloride (KH070404, Jiangsu Heng Rui Medicine Co., Jiangsu, China), injection of vecuronium (208609, Nanjing Organon Pharmaceutical Co., Nanjing, China), isoflurane (535335U, Shanghai Yapei Pharmaceutical, Shanghai, China), and sevoflurane (070530, Jiangsu Heng Rui Medicine Co.).

### Research subjects

Thirty pediatric patients above 12 kg for elective surgery of grades I and II, in accordance to the qualifications of American Anesthetic Association, were recruited, including 12 boys and 18 girls with body weights between 12 and 34 kg, ages ranging from 1 to 7.7 years, and heights between 78 and 138 cm, respectively. The duration of surgery was between 114 and 490 minutes (197 ± 82 min). The study was approved by the Institutional Review Board prior to the subjects being enrolled and pre-operative informed consent was obtained from parents of all the participants. The exclusion criteria were the following: allergic to propofol, history of anesthetic complications, liver and kidney impairment, hematological disease, metabolic disorders, inherent tracheal problem, inability to cooperate for questionnaires, anticipated hemodynamic instability during surgery, subjects requiring specific or large quantity of infusion, and expected shorter duration of surgery (less than 1 hour).

### Drug administration

Pre-operative preparations included routine fasting before surgery and 10 minutes of resting in supine position after entering the operation room. Vital parameters were measured using the hypertension multi-function monitor, and the degree of sedation was monitored. The unilateral peripheral vein was used for injection of sodium lactate and Ringer's solution at 10-15 mL·kg^-1^·h^-1^. Anesthetics was induced and maintained with the continuous venous infusion of propofol at 1.0 ug·mL^-1^, followed by midazolam at 0.01 mg·kg^-1^, ketamine hydrochloride at 2 mg·kg^-1^, and vecuronium at 2 mg·kg^-1^. After 5 minutes, tracheal intubation and intermittent ventilation under positive pressure were performed to maintain the carbon dioxide partial pressure at 35 to 45 mmHg. For anesthesia maintenance on basis of the age and body weight, the TCI-III system for propofol (Pediatric mode) was set with targeted plasma concentration of 1 to 4 μg·ml^-1 ^for controlled infusion. Dinitrogen oxide-isoflurane- oxygen or both-sevoflurane, was used for anesthetic maintenance. For the postoperative monitoring, anesthetic gas was shut off after suturing during surgery and the TCI pump was adjusted to concentration for recovering consciousness when the expired concentration reached zero.

### Observational indices

Observational indices were selected on basis of measurements related to the general life information, adverse reactions, and drug-associated effects. During the president study the age, body weight, height, sex, admission number, anesthetic drug combination, surgical procedure, and demographic characteristics of patients were recorded. Vitals signs were observed, including electrocardiography, oxygen saturation, non-invasive blood pressure, electrocardiograph - bispectral index, target concentration of propofol at each time point of blood sampling, total dosage of propofol for anesthetization, total time of propofol usage, and adverse reactions, including brachycardia, heart rate < 50 bpm, low blood pressure systolic pressure < 85 mmHg, or systolic pressure lower than 30% or more of the basal value.

### Collection of plasma samples

The initial target concentration for sedation with propofol under TCI was 1.0 μg/ml^-1 ^and 2 to 4 target concentrations were set accordingly to surgical status during anesthetization. When a target concentration shown by the equipment remained constant 5, 10, 20, and 30 minutes after the adjustment, 1.5 to 2 ml of peripheral venous blood sample was collected from another site and placed in the tube with anti-coagulant EDTA. Samples were stored in 4°C refrigerator after mixing. Within 2 hours, it was centrifuged under room temperature at the rotation speed of 400 × *g *for 10 minutes. Plasma was stored in -80°C till further analysis.

### Handling and measuring of plasma samples

The plasma concentration of propofol was measured with high-performance liquid-phase chromatography and fluorescent method using Thymol as an internal standard and acetonitrile as the protein precipitator. The analytical column used was Zorbax Eclipse C18 (250 mm × 4.6 mm, 5 μm) at a column temperature of 20-24°C. The mobile phase consisted of acetonitrile and water (70:30) at pH 4.0 adjusted with glacial acetic acid and was pumped at a flow-rate of 1.0 mL· min ^-1^. The excitation wavelength was set at 276 nm and emission at 310 nm. 100 uL of plasma sample was accurately acquired and placed in the tube. 200 uL of acetonitrile with internal standard was added as protein precipitator before placing it on the vortex mixer for 30 seconds and then centrifuged for 10 minutes at 1300 × *g*. 10 uL supernatant was obtained and loaded into the high-performance liquid-phase chromatography and fluorescent instrument for measuring plasma concentration of propofol. The assessment results for the methodology showed the following: the linear range of measurement fell between 19 and 9630 ng·mL^-1 ^(*r *= 0.9999, *n *= 8) and the lowest measuring concentration was 0.38 ng·mL^-1^. The recovery rate of concentrations at 39, 770, and 7704 ng·mL^-1 ^were 100, 103 and 101 after storing 6 and 24 h in room temperature, freezing for 30 days and exchanging between freezing and thawing 3 times (n = 5). The variable coefficient of daily precision was ≤1.0%, and the variable coefficient between dates was ≤3.75%. Intrinsic compounds and preoperative or surgical medicines did not affect the measurement of propofol concentration in the plasma of healthy subjects.

### Data processing and statistical analysis

Data were analyzed using SPSS 11.5 software and presented as mean ± standard deviation. The performance assessment of TCI system was performed as described by Varvel et al. [10]. The variance between the actual measured drug concentration in plasma and the target concentration at corresponding time point was presented in percentage of performance error (PE): PE%= (concentrationij - target concentrationij )/target concentrationij × 100%, reflecting the precision of the measuring system. The bias of system was presented by the medium value of PE (MDPE): MDPEi = median {PEij; j = 1,..., Ni }, describing whether the performance of the system was lower or higher than expected. The precision of the system was presented by the medium value of absolute value of performance error (MDAPE): MDAPEi = median {|PE|ij, j = 1,..., Ni }, indicating the inaccuracy of the control system. The degree of Wobble in the system was presented by the medium value of absolute deviation: Wobble = median {|PEij -MDPEij|, j = 1,..., Ni }, representing the variability of performance error.

### Performance assessment of target control infusion system for propofol

Currently, most references were used including bias (MDPE), precision (MDAPE), and the degree of Wobble in the system as assessment indices for the performance of TCI system. It is generally believed that MDPE <15% and MDAPE<30% indicated satisfaction in clinical application [10-12].

## Results

**Figure 1:**
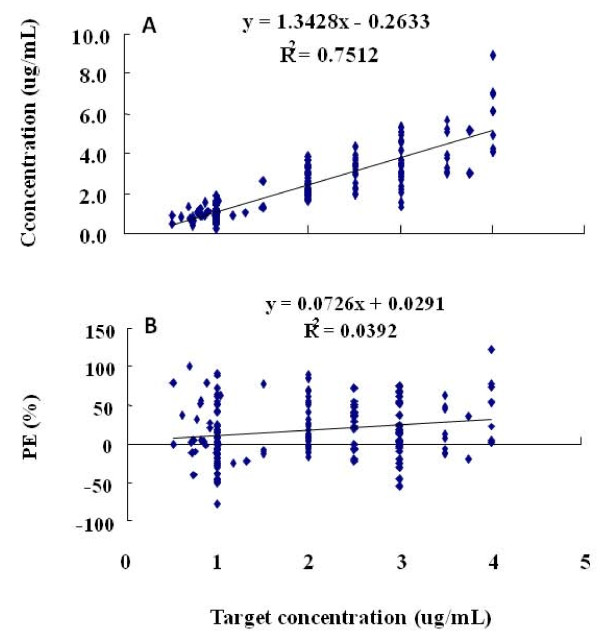
**Predicted concentrations of Propofol in 1<year< 8 by SLOG TCI-III (Pediatric) system (Figure 1A), and performance error (PE%) against the calculated concentrations in 1<year< 8 by SLOG TCI-III (Pediatric) system (Figure 1B)**.

Blood sampling and results of analysis by high performance liquid chromatography are shown in Table 1. Comparative analysis was performed on the targeted drug concentrations in plasma after drug administration by TCI-III "Pediatric" system and the actual measured drug concentrations in 180 collected blood samples. Linear regression was drawn for concentration and target concentration, where the linear equation turned out to be: concentration = 1.3428 target concentration - 0.2633 (*r *= 0.8667) (Figure 1A). It suggested a close linear relationship between concentration and target concentration. Linear regression was drawn again for target concentration and performance error (PE) and the equation was calculated as: PE = 0.0726 target concentration + 0.0291(*r *= 0.1980) (Figure 1B).

**Table 1:** The relationship between target concentration and actual measured concentration ($\bar{x}$ ± SD)

Target Conc.(μg·mL^-1^)	Measured Conc.(μg·mL^-1^)	Performance error(PE)(%)
0.51	0.91	0.79

0.52	0.52	0.00

0.61	0.84	0.37

0.69	1.38	1.01

0.71	0.73	0.03

0.72	0.64	-0.12

0.73	0.6 ± 0.24 (n = 2)	-17.72 ± 32.81(n = 2)

0.75	0.67	-0.11

0.78	1.04	0.33

0.81	1.23	0.52

0.82	1.07 ± 0.3 (n = 2)	31.06 ± 36.68(n = 2)

0.84	0.88	0.04

0.87	0.86	-0.01

0.88	1.57	0.78

0.91	1.13 ± 0.04 (n = 2)	0.24 ± 0.004 (n = 2)

0.98	0.86	-0.12

1	1.02 ± 0.38(n = 52)	1.67 ± 37.84(n = 52)

1.01	1.02	0.01

1.02	1.66	0.63

1.17	0.87	-0.26

1.32	1.04	2.5-0.21

1.5	1.67 ± 0.65(n = 4)	11.35 ± 43.62(n = 4)

2	2.53 ± 0.63(n = 35)	26.62 ± 31.52(n = 35)

2.5	3.2 ± 0.69(n = 18)	27.83 ± 28.79(n = 18)

3	3.5 ± 1.07(n = 29)	16.82 ± 35.61(n = 29)

3.5	4.14 ± 1.04(n = 8)	18.17 ± 29.62(n = 8)

3.75	4.07 ± 1.48(n = 2)	8.53 ± 39.39(n = 2)

4	5.83 ± 1.66(n = 9)	45.81 ± 41.54(n = 9)

**Figure 2:**
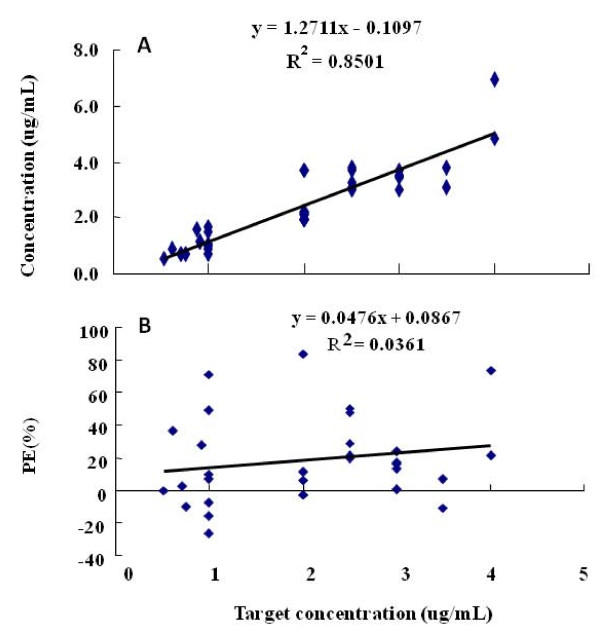
**Predicted concentrations of Propofol in 1<year< 3 by SLOG TCI-III(Pediatric)system (Figure 2A), and performance error (PE%) against the calculated concentrations in 1<year< 3 by SLOG TCI-III (Pediatric) system (Figure 2B)**.

**Figure 3:**
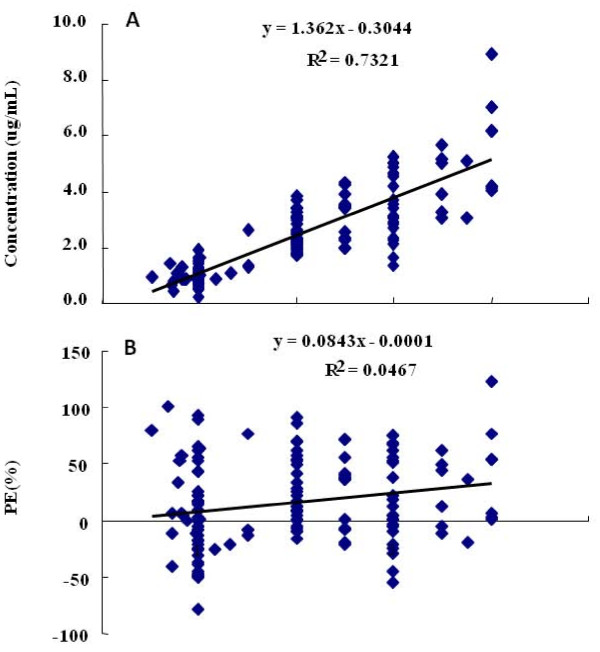
**Predicted concentrations of Propofol in 3<year < 8 by SLOG TCI-III (Pediatric) system (Figure 3A), and performance error (PE%) against the calculated concentrations in 3<year < 8 by SLOG TCI-III (Pediatric) system (Figure 3B)**.

180 blood samples were analyzed accordingly to age differences: 1<yr<3 and 3<yr<8 (Table 2, Figure 2, 3). The values of MDPE, MDAPE, and Wobble of children between 1 and 8 years old were 10%, 22%, and 24%, respectively. The values of MDPE, MDAPE, and Wobble of children between 1 and 3 years old were 15%, 17%, and 14%, respectively. The values of MDPE, MDAPE, and Wobble of children between 3 and 8 years old were 9%, 25%, and 28%, respectively. The values of MDPEs at all age groups using TCI-III "Pediatric" system were less than 15% and MDAPEs were less than 30%, of which all fell within acceptable clinical range.

**Table 2:** The performance status of SLOG/TCI-III (Pediatric) system in age groups

Age group	1<year< 3	3<year < 8	1<year< 8
Concentration(Y)and Target concentration(X) Linear Regression:	y = 1.2711 × - 0.1097 (*r *= 0.9220)	y = 1.362 × - 0.3044 (*r *= 0.8556)	y = 1.3428 × - 0.2633(*r *= 0.8667)
PE(Y) Target concentration (X) Linear Regression:	y = 0.0476 × + 0.0867(*r *= 0.1900)	y = 0.0843 × - 0.0001 (*r *= 0.2161)	y = 0.0726 × - 0.0291(*r *= 0.1980)
MDPE (%)	14.89	8.75	9.58
MDAPE (%)	16.69	24.58	21.77
Wobble (%)	14.25	27.69	23.62

**Abbreviations: **PE: performance error; MDPE: medium value of performance error; MDAPE: the medium value of absolute value of performance error; Wobble: medium value of absolute deviation.

## Discussion

Pharmacokinetic parameters of propofol vary with age, sex, body weight, disease condition, and usage of concomitant drug. The central compartment is larger in volume and the clearance rate is higher in children. For children of age 3 or above, the distributional volume and clearance rate require adjustment according to the body weight. For children under 3-years-old, pharmacokinetic parameters correspond positively with body weight, in which the clearance rates of the central compartment and entire body are much higher than those of older children [13,14]. Therefore, the present study investigated alterations in further subdivided groups betwe,en >3-years-old and <3-years-old.

The performance of the TCI system depends primarily on the degree of closeness achieved between target concentration and concentration in the plasma. However, TCI system may hardly maintain the equilibrium between measured concentration and the target concentration, due to the variations between individuals or groups. The target concentration could be adjusted according to clinical need, if the measured and target concentrations were in parallel relationship. The range of measured concentrations of propofol was wider. The performance error demonstrated a positive relationship with target concentration for propofol, similar to the previous finding [15]. The MDPE of TCI-III Pediatric system showed that the measured concentrations of propofol in blood were generally higher than the target. It implies that the TCI-III pediatric system underestimated the drug concentration in blood. Clinical application of those TCI-III systems could cause a deeper anesthesia than expected, leading to increased hemodynamic fluctuation and other risks of excess anesthesia.

The present study is the initial one to evaluate the application of SLOG TCI-III system in pediatric surgery. Results showed that MDPEs and MDAPEs at all age groups of children were within normal range, suggesting that the TCI-III system could effectively achieve the target goal in the selected patients. The degree of Wobble in the system was slightly higher in children of 1-3 and 3-8 years old, respectively, probably due to the wide variation in pharmacokinetic characteristics of propofol in children.

The evaluation of SLOG/TCI-III system on target-controlled infusion of propofol has been performed in clinic, of which measurements were clinical parameters. For example, the simultaneous closed-loop anesthesia control system was clinically evaluated [16]. The effect of propofol and remifentanil combinations was modeled and compared on basis of clinical measurements [17,18]. The present study indicates the needs of system-specific biomarkers by translational medicine. Dr Marincola recently emphasized the importance and need to increase the understanding of human pathophysiology and use surrogate biomarkers to early assess efficacy of intervention [19]. The great challenge is how to translate clinical questions and problems from the "bed" to "bench" by using advanced biotechnologies [20]. Clinical and translational science provides a new approach to develop and validate disease-specific or intervention-associated biomarkers [21-23]. It is possible to integrate clinical information with bioinformatics driven from plasma proteomics and discover new drug-specific biomarkers for the further evaluation [24-26].

In conclusion, clinical application of the target concentration could be appropriately adjusted by the anesthesiologist, according to the need of degree of anesthetization, in order to overcome the differences in pharmacokinetics and pharmacodynamics between individuals. There is a need to further evaluate the performance of TCI-III in a large population of patients. The performance of TCI-III system seems to be within the accepted limits for clinical practice in children. It could make the TCI-III system much safer and convenient to use.

## Competing interests

The authors declare that they have no competing interests.

## Authors' contributions

YWH, WXD, YYF designed experiments, reviewed data, supervised assay conduct and wrote sections of the manuscript; GHB and FQW provided many patient blood samples; CB, LJ helped design experiments, designed and performed all statistical analyses and wrote sections of the manuscript. All authors read and approved the final version of the manuscript.
